# The quest for the best: The impact of different EPI sequences on the sensitivity of random effect fMRI group analyses

**DOI:** 10.1016/j.neuroimage.2015.10.071

**Published:** 2016-02-01

**Authors:** Evgeniya Kirilina, Antoine Lutti, Benedikt A. Poser, Felix Blankenburg, Nikolaus Weiskopf

**Affiliations:** a Neurocomputation and Neuroimaging Unit, Department of Psychology and Educational Science, Free University Berlin, Habelschwerdter Allee 45, 14195 Berlin, Germany; b Department of Neurophysics, Max Planck Institute for Human Cognitive and Brain Sciences, Stephanstrasse 1a, 04103 Leipzig, Germany; c Laboratoire de Recherche en Neuroimagerie, Département des Neurosciences Cliniques, Centre Hospitalier Universitaire Vaudois, Chemin de Mont Paisible 16, 1011 Lausanne, Switzerland; d Wellcome Trust Centre for Neuroimaging, UCL Institute of Neurology, University College London, 12 Queen Square, London WC1N 3BG, UK; e Department of Cognitive Neuroscience, Maastricht Brain Imaging Centre, Faculty of Psychology & Neuroscience, Maastricht University, PO Box 616, 6200MD Maastricht, The Netherlands

**Keywords:** fMRI sensitivity, Echo planar imaging (EPI), 3D EPI, Multi-echo EPI, Inter-subject variability, Random effects analysis

## Abstract

We compared the sensitivity of standard single-shot 2D echo planar imaging (EPI) to three advanced EPI sequences, i.e., 2D multi-echo EPI, 3D high resolution EPI and 3D dual-echo fast EPI in fixed effect and random effects group level fMRI analyses at 3 T. The study focused on how well the variance reduction in fixed effect analyses achieved by advanced EPI sequences translates into increased sensitivity in the random effects group level analysis. The sensitivity was estimated in a functional MRI experiment of an emotional learning and a reward based learning tasks in a group of 24 volunteers. Each experiment was acquired with the four different sequences. The task-related response amplitude, contrast level and respective t-value were proxies for the functional sensitivity across the brain. All three advanced EPI methods increased the sensitivity in the fixed effects analyses, but standard single-shot 2D EPI provided a comparable performance in random effects group analysis when whole brain coverage and moderate resolution are required. In this experiment inter-subject variability determined the sensitivity of the random effects analysis for most brain regions, making the impact of EPI pulse sequence improvements less relevant or even negligible for random effects analyses. An exception concerns the optimization of EPI reducing susceptibility-related signal loss that translates into an enhanced sensitivity e.g. in the orbitofrontal cortex for multi-echo EPI. Thus, future optimization strategies may best aim at reducing inter-subject variability for higher sensitivity in standard fMRI group studies at moderate spatial resolution.

## Highlights

• We compared the sensitivity of standard and three advanced EPI sequences in group level fMRI analysis.
• Advanced EPI methods increased the sensitivity in the fixed effect group analyses.
• All EPI methods provided similar sensitivity in random effect group analysis.
• Inter-subject variance determined the sensitivity of the random effect analysis.
• Future sequence optimization best aim at reducing inter-subject rather then intra-subject variance.

## Introduction

The optimization of functional MRI (fMRI) pulse sequences is based on a well-balanced trade-off between several requirements. Typical fMRI studies benefit from whole brain coverage and high spatial resolution on the one hand, and high temporal resolution as well as high sensitivity and specificity on the other hand. To achieve high sensitivity to the blood-oxygen level dependent (BOLD) effect across the entire brain, a compromise between high general BOLD sensitivity due to pronounced *T*_2_*-weighting and reduced susceptibility-related signal dropouts in frontal and deep brain regions (Weiskopf et al., 2007a) has to be achieved. Furthermore, physiological noise contributions due to e.g. heart pulsation, respiration and head motion need to be minimized (Birn et al., 2006, Chang and Glover, 2009, Glover et al., 2000, Krüger and Glover, 2001). Finally, to reach high sensitivity in random effects group level analysis, inter-subject variability needs to be minimized, which can be caused by inter-individual differences in BOLD response amplitudes or anatomical variability.

In fMRI at 3 T a reasonable and most frequently used trade-off for data acquisition is based on slice-selective gradient-echo echo planar imaging (2D EPI) with a repetition time *T*_R_ ~ 2 s, an echo time *T*_E_ ~ 30 ms and an isotropic resolution of 3 mm. This approach proved to provide good BOLD sensitivity and robust performance in most brain regions. However, it has only moderate temporal and spatial resolution and suffers from dropouts in the orbitofrontal cortex (OFC), and deep brain regions such as the amygdala (Morawetz et al., 2008, Weiskopf et al., 2006, Weiskopf et al., 2007a, Deichmann et al., 2002, Deichmann et al., 2003).

To overcome these limitations, recently novel EPI approaches have been developed capitalizing on progress in k-space acquisition trajectories, parallel imaging and simultaneous excitation (Lutti et al., 2013, Poser et al., 2006, Poser et al., 2010, Feinberg et al., 2010, Setsompop et al., 2012). This study assesses the benefits of three advanced EPI sequences employing different combinations of parallel imaging, 3D k-space trajectories and multi-echo acquisitions (Lutti et al., 2013, Poser et al., 2006, Poser et al., 2010).

The first EPI sequence is based on 2D single-shot multi-echo EPI (2D ME EPI) which acquires images at multiple *T*_E_ values after each spin excitation (Poser et al., 2006, Posse et al., 1999). The images at different *T*_E_ values are combined in an optimally weighted sum to achieve a high sensitivity in every brain region. In addition images with multiple contrasts provide the possibility to remove non-BOLD like noise components (Kundu et al., 2012). The 2D ME EPI was shown to increase the BOLD sensitivity and decrease physiological noise levels as compared to standard 2D EPI.

The second tested approach uses high-resolution 3D EPI (3D HR EPI) acquisitions. At high spatial resolution thermal noise is the dominant source of instability in fMRI time-series (Triantafyllou et al., 2005) and multi-shot 3D EPI acquisitions have been shown to yield higher image signal-to-noise ratio (SNR) than their 2D counterparts (Lutti et al., 2013, Poser et al., 2010). Previous work suggests that spatial smoothing of high resolution data to lower resolution exceeds the expected BOLD sensitivity gain (Triantafyllou et al., 2006). Moreover, a higher resolution reduces susceptibility related signal loss (Weiskopf et al., 2007a, Merboldt et al., 2001).

The third acquisition approach employs a 3D dual-echo EPI (3D DE EPI) sequence (derived from Lutti et al., 2013), which combines the advantages of 2D ME EPI and 3D HR EPI. This sequence allows for *T*_R_ shortening while maintaining the spatial resolution at a minimal cost in BOLD sensitivity due to the higher SNR provided by 3D acquisitions.

These advanced EPI methods have shown increased sensitivity in single subject fMRI experiments, using fixed-effects analysis, as compared to standard 2D EPI (Lutti et al., 2013, Poser et al., 2006). However, the assessment was limited to fixed effects analysis in small populations and specific experimental designs. Since the performance may depend on multiple factors such as area of interest, experimental design, subject anatomy and physiological noise, the above-mentioned results may not be generally extrapolated to other domains. Furthermore, it is unclear how the variance reduction provided by advanced sequences at the single subject level will translate into an increased sensitivity at the second level group analysis, where inter-subject variance is believed to dominate (Friston et al., 2005).

Thus, we specifically assess the performance of different EPI pulse sequences for fMRI group studies under well-defined and comparable conditions. The functional sensitivity of the standard and three advanced EPI sequences is determined for multiple brain areas in random effects group analysis. Inter- and intra-subject variability is explored in well-established fMRI of emotional and reward learning tasks, which induce functional activation in different brain regions. Finally, we provide guidelines for an informed choice of the optimum EPI sequence for a specific neuroimaging application, based on experimentally obtained empirical data.

## Methods

The sensitivity of four EPI sequences was compared in the random effects group level analysis on the same subject population and using the same experimental paradigm. The imaging parameters of one standard and three advanced EPI sequences (see the Data acquisition section and Table 1 for details) were aligned as far as possible to provide a realistic comparison between sequences. The subject population described in detail in the Subjects section participated in four fMRI runs, each recorded by one of the four EPI sequences. To control for potential habituation, the order of the four EPI sequences was pseudo-randomized and completely counterbalanced over the subject population. The experimental paradigm described in detail in the Tasks section was designed to be as similar as possible to tasks typically utilized in fMRI experiments. Importantly, the chosen tasks induce robust BOLD responses in primary cortical areas, subcortical areas and brain areas prone to susceptibility artifacts such as the amygdala and the OFC.

### Data acquisition

All fMRI measurements were performed on a 3 T TIM Trio (Siemens, Erlangen, Germany, Software VB17) MRI scanner, equipped with a 32-channel radio-frequency (RF) receive head coil and body RF transmit coil.

#### Standard EPI (2D EPI)

The vendor's standard 2D EPI sequence with *T*_R_/*T*_E_ = 2 s/30 ms, field of view FOV = 192 mm, matrix size = [64 × 64], fat saturation pulse, an excitation flip angle FA = 70°, a readout bandwidth BW = 2232 Hz/Px and echo spacing ES = 0.53 ms was used as standard sequence. Thirty-seven 3 mm thick slices oriented along the anterior commissure (AC)–posterior commissure (PC) anatomical axis with an inter-slice gap of 20% were recorded in interleaved order, using the anterior–posterior (A–P) axis as the phase-encoding (PE) direction. Parallel imaging with an acceleration factor AF = 2 was used along the PE direction. Images were reconstructed using the generalized autocalibrating partially parallel acquisition (GRAPPA) method (Griswold et al., 2002) using 24 reference lines.

#### Multi-echo (2D ME EPI)

The 2D ME EPI sequence (Poser et al., 2006) acquired five images with *T*_R_ = 2.5 s/*T*_E1_–*T*_E5_ = (7.4 ms, 17.2 ms, 27 ms, 37 ms, 47 ms), FOV = 192 mm, matrix size = [64 × 64], fat saturation pulse, FA = 70°, BW = 2520 Hz/Px and ES = 0.5 ms. Thirty-seven 3 mm thick slices oriented along the AC–PC axis with an inter-slice gap of 20% were recorded in interleaved order, using the A–P axis as the PE direction. Parallel imaging with AF = 3 and partial Fourier (PF) with 6/8 coverage of k-space in the PE direction were applied. Images were reconstructed using the GRAPPA method using 24 reference lines.

#### Fast (3D DE EPI)

A dual-echo 3D EPI pulse sequence (derived from Lutti et al., 2013) with an isotropic nominal resolution of 3 mm, *T*_R_/*T*_E1_, *T*_E2_ = 50 ms/(15.9 ms, 34.4 ms), FOV = 192 mm, matrix size = [64 × 64 × 60], BW = 2367 Hz/Px and ES = 0.56 ms was used for fast fMRI imaging. Parallel imaging with AF = 2 along the PE (A–P axis) and AF = 3 along the partition-encoding direction (slow second PE direction along left–right) was used. Slab selective excitation was used with sagittal slab orientation and water-selective binomial RF pulses and FA = 15°. A linear increment of the partition-encoding was used and a single partition-encoding step was acquired per *T*_R_. With these parameters a volume repetition time *T*_Rvol_ = 1000 ms was achieved. Images were reconstructed using the GRAPPA method. The GRAPPA reconstruction kernel was estimated from a fully sampled volume acquired at the beginning of each fMRI time series using a multi-shot segmented k-space scheme.

#### High resolution (3D HR EPI)

A 3D EPI sequence (Lutti et al., 2013) with isotropic nominal resolution of 2 mm, *T*_R_/*T*_E_ = 70 ms/33 ms, FOV = 192 mm, matrix size = [96 × 96], FA = 20° and BW = 1408 Hz/Px was used for high spatial resolution fMRI imaging. Sixty-four axial slices were recorded. A fat saturation pulse with a FA = 110° was applied. Parallel imaging with AF = 2 along the PE (A–P) and AF = 2 along the partition-encoding (inferior–superior) direction was used. Oversampling of 6% was used in the partition-encoding direction to avoid wrap-around artifacts due to imperfect slab excitation. A linear increment of the partition-encoding was used and a single partition-encoding step was acquired per *T*_R_. With these parameters a *T*_Rvol_ = 2520 ms was achieved. Images were reconstructed using the GRAPPA method. The GRAPPA reconstruction kernel was estimated from a fully sampled volume acquired at the beginning of each fMRI time series using a multi-shot segmented k-space scheme.

Dual-echo gradient echo (GE) B_0_ field map images (*T*_E1_/*T*_E2_ = 10 ms/12.46 ms) were acquired at the beginning, and *T*_1_-weighted anatomical images (MPRAGE *T*_R_/*T*_E_/*T*_I_/FA/BW = 2300 ms/3.03 ms/900 ms/9°/130 Hz/Px, 1 × 1 × 1 mm^3^ resolution) at the end of the scanning session.

The participant's heart rate was recorded with the scanner's internal optical plethysmograph attached to the left index finger. Respiration related thoracic movements were detected by a pneumatic respiration belt placed around the abdomen close to the lower ribs and recorded by an ExG amplifier (Brain Products GmbH, Gilching, Germany).

### Subjects

Twenty-four healthy volunteers (13 females, mean age ± standard deviation = 29.2 ± 5.8 years, all right-handed) participated in the study. All subjects gave written informed consent in compliance with a protocol approved by the local ethics committee. None of the subjects reported a history of neurological diseases or was undergoing pharmacological treatment.

### Tasks

**Fig. 1:**
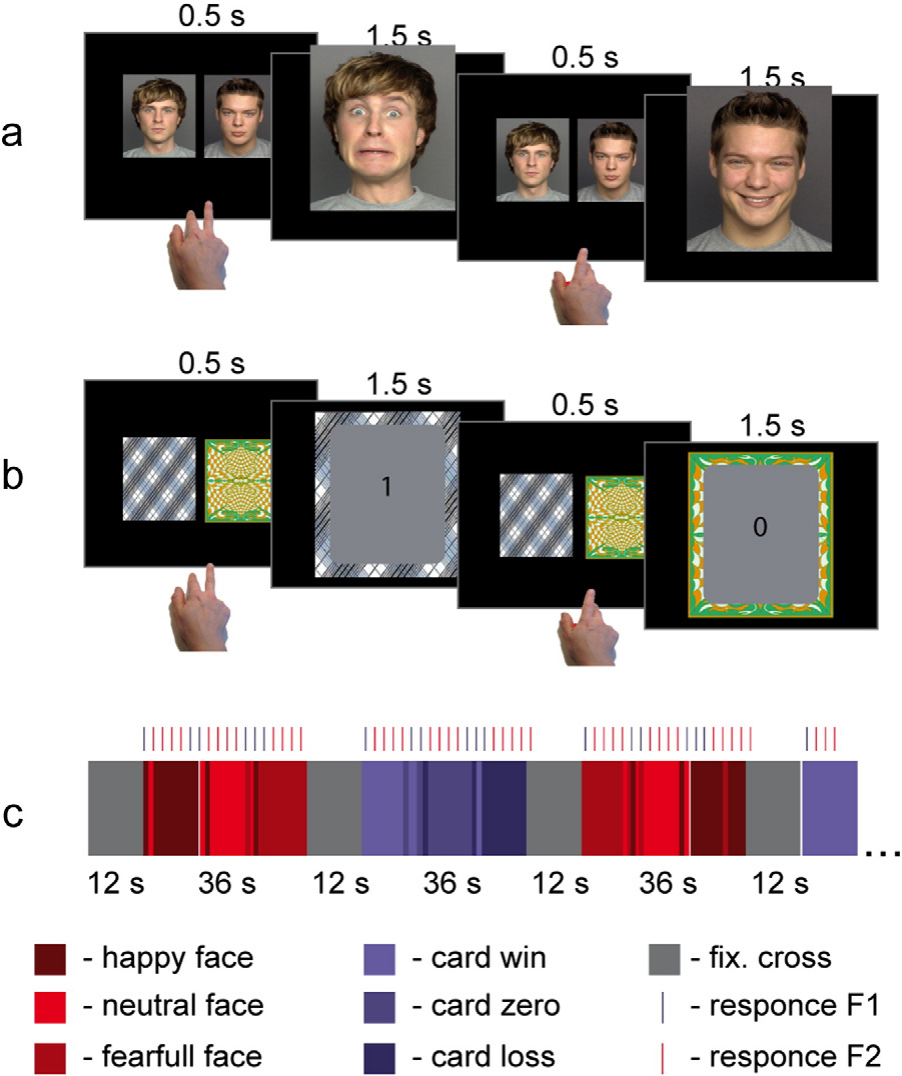
(a) Emotional learning (EL) task, (b) reward based learning (RBL) task, and (c) experiment time line. Blocks of EL and RBL tasks were presented in interleaved order, separated by rest periods in which a fixation cross was presented.

The subjects performed two types of tasks, i.e., emotional learning (EL) and reward based learning (RBL), as illustrated in Fig. 1. Tasks were presented in 36 s long blocks in interleaved order and were separated by 12 s rest periods, during which a fixation cross was presented in the middle of the screen. Each 36 s block contained 18 trials of the EL or RBL task.

At the beginning of each EL trial photos of two persons with neutral facial expressions were presented to the subject for 500 ms. In the RBL task pictures of two facedown card decks were presented instead of the faces. Subjects were instructed to choose the right or the left person (or right or left deck in RBL task) by clicks on a right or left response button, respectively. Then in the EL task the selected person was presented for 1500 ms in the middle of the screen with happy, neutral or fearful facial expression. In the RBL task the card from the selected deck was then turned over and presented for 1500 ms indicating the win/loss of 1, 0 or − 1 points for the trial. In the EL task the subject won one point if the selected person's facial expression was happy, lost a point when the facial expression was fearful and received zero points if the facial expression was neutral. The account update was presented to the subject for 800 ms, in the middle of the screen 6 s after the end of each block. Photos of different persons and different decks of cards were used in each block in order to minimize habituation.

The facial expressions of the right and left persons as well as the gains of the left and right card decks were predefined. Each of the 36 s blocks contained three 12 s phases in which: 1) both persons (or decks) were neutral (or were zero gain in card condition); 2) both persons were fearful (both decks bring loss); and 3) one person was consistently happy, another was consistently fearful (one deck brings win another loss). In order to avoid complete predictability 2 to 4 inconsistent trials were added to each block. The objective of the game was to get as many points as possible. Thirty second baseline resting periods (fixation cross presented to the subject) were recorded in the beginning and in the end of each run.

Subjects were endowed with 15 Euro at the beginning of the experiment. Depending on their performance over entire experiment they could either win or lose up to 5 Euro, resulting in a minimal/maximal final reimbursement of 10/20 Euro.

The paradigms were implemented in the software package Presentation (Version 7, Neurobehavioral Systems). During fMRI, subjects were lying supine in the MRI scanner and observed stimuli on the screen mounted on the scanner's bore opening through a 90° mirror fixed to the RF head coil. A fiber optic response device with two buttons was used to register the subjects' responses. The response was given with the right hand index and middle fingers.

Subjects performed four 7 minute 24 second runs, each run containing 4 blocks of EL and 4 blocks of RBL tasks. The order of pulse sequences was fully counterbalanced across the 24 subjects.

### Data analysis

The data were analyzed using SPM8 software (http://www.fil.ion.ucl.ac.uk/spm/) and custom made programs in MATLAB (The MathWorks Inc., Natick, MA, USA).

#### Data pre-processing

2D EPI and 2D ME EPI images were corrected for the different timing of the slice acquisition by temporal interpolation to the acquisition time of the slice in the center of the volume using the standard method in SPM8. The 2D EPI, 3D HR EPI and 3D DE EPI images were realigned and unwarped, using the Realign&Unwarp function of SPM8 and the recorded B_0_ field maps. The 2D ME EPI images were only realigned using the Realign function of SPM8 and motion parameters obtained from the images recorded at *T*_E_ = 27 ms (the contrast of this images closely corresponded to the contrast of standard 2D EPI images). 3D DE EPI images corresponding to two *T*_E_ values were combined as a simple sum. 2D ME EPI images corresponding to five different *T*_E_ values were combined using the ‘parallel acquired inhomogeneity desensitized’ (PAID) algorithm (Poser et al., 2006). For PAID the 15 volumes recorded in the beginning of the run were smoothed with a Gaussian filter (4 mm full width at half maximum (FWHM)) and used to estimate the voxel dependent temporal signal-to-noise ratio (*t*SNR*_T_*_*_E_*_), for each *T*_E_. The images corresponding to five different *T*_E_ values were then combined as described below:

(1) $$S_{\mathrm{comb}}^{\mathrm{PAID}}\left(t\right)=\sum_{T_{E}}S_{T_{E}}\left(t\right)\cdot T_{E}\cdot tSNR_{T_{E}}$$

where *S_T_*_*_E_*_ (*t*) is the intensity of an image recorded at time point *t* corresponding to a particular *T*_E_.

Then all images were bias corrected by segmenting the first image and applying the bias field obtained during the segmentation procedure to all images. Images were then normalized to the Montreal Neurological Institute (MNI) template. The MNI normalization was performed based on the anatomical *T*_1_-weighted image, which was co-registered to the mean time-series EPI image. For 2D ME EPI the mean image corresponding to *T*_E_ = 27 ms was used. 3D HR EPI images were interpolated to the isotropic resolution of 3 mm during the normalization step. The normalization procedure included a 12-parameter affine transformation in the first step and non-linear deformation defined by a linear combination of 3D discrete cosine transform (DCT) basis functions in the second step. Trilinear interpolation was used for image resampling. Finally normalized images from all EPI sequences were smoothed with a Gaussian kernel of 8 mm FWHM.

#### Physiological de-noising

To reduce the impact of physiological noise on the temporal SNR and BOLD sensitivity, two different methods were used.

For the first method, a set of 20 physiological regressors were generated based on cardiac and respiratory traces recorded with the respiration belt and pulse oximeter, and head motion traces estimated by the realignment procedures (Hutton et al., 2011). The set included the first, second and third harmonics of cardiac and respiratory phases, respiratory volume per time unit and cardiac volume per time unit and six motion parameters.

For the second method a set of 16 auxiliary physiological regressors was generated using the CompCor method (Behzadi et al., 2007). Briefly, this algorithm first generated cerebral spinal fluid (CSF) and white matter (WM) masks based on the segmented *T*_1_-weighted images for each participant. These masks were smoothed with a Gaussian kernel of 8 mm FWHM and cut at a threshold of 0.95 to provide more conservative masks. Time courses of the first five principal components extracted from the EP images masked with CSF mask and five principal components extracted from the EP images masked with WM mask were used as the first 10 auxiliary regressors. At this stage we used the EP images prior to smoothing and normalization. Time courses of six motion parameters extracted in the re-alignment step were used as six additional regressors.

For all four pulse sequences we obtained better results for the second method as compared to the first (results are not shown). Therefore, only the second (CompCor) method was applied for physiological de-noising in the final analysis.

#### Statistical analysis

Pre-processed images of each subject and all four EPI sequences underwent a fixed effects general linear model (GLM) analysis. The GLM included seven functional (face happy, face neutral, face fearful, card win, card loss, card zero, account update) predictors and the 16 nuisance regressors accounting for physiological noise (generated by CompCor method as described in the Physiological de-noising section). Functional predictors were simulated by convolution of standard SPM hemodynamic response function with boxcar functions corresponding to onsets of the respective conditions. Analyses were performed for two contrasts. The first contrast ‘tasks vs. rest’ included six functional predictors (all faces and all cards) contrasted to the baseline (fixation cross). The corresponding contrast vector was *c* = [1 1 1 1 1 1]. The second contrast ‘EL vs. RBL’ contrasted three face predictors with three card predictors with a contrast vector *c* = [1 1 1 − 1 − 1 − 1]. The two contrast maps for each volunteer were entered in the second level random effects analysis. The following random effects group analysis estimated the contrast maps, variance maps and t-maps for the group from the previous single subject contrasts. The t-maps were thresholded at an uncorrected voxel-wise significance level of p < .001. The correction for multiple comparisons was performed on the cluster level. Activation clusters were regarded as significant if they were larger than 10 voxels and reached an uncorrected voxel-wise significance level of p < .001 and a cluster whole brain family wise error (FWE)-corrected level of p < 0.05. The resulting random effects analysis t-maps obtained for four EPI sequences were used as proxies for the sensitivities of four EPI methods to functional activation.

Second level random effects t-value *t*^(2)^ for the particular contrast *c* is provided by the ratio

(2) $$t^{\left(2\right)}=\frac{\langle c^{T}\beta^{\left(1\right)}\rangle }{\sqrt{c^{T}\mathrm{var}\left\{\beta^{\left(1\right)}\right\}c}}\text{,}$$

where *β*^(1)^ is a vector of BOLD-effect amplitudes estimated in the single subject fixed effects analyses and the angular brackets denote group averaging. Differences in t-values obtained for four different EPI sequences could be attributed either to the differences between the numerators (mean contrast values) in Eq. 2 or to the differences between the denominators (parameter variance) of Eq. 2. In order to disentangle the two driving factors for differences between sequences, a separate analysis of the parameter sizes and parameter variances was performed for four sequences. The variance matrix var{*β*^(1)^} in the denominator of Eq. 2 is a variance of *β*^(1)^-estimates, which can be expressed as a sum of inter-subject and intra-subject variance contributions (Friston et al., 2005):

(3) $$\mathrm{var}\left\{\beta^{\left(1\right)}\right\}=C^{\left(2\right)}+X^{\left(1\right)-}C^{\left(1\right)}X^{\left(1\right)-T}\text{,}$$

where *C*^(2)^ is a true inter-subject variance of parameters *β*^(1)^ and term *X*^(1) −^*C*^(1)^*X*^(1) − *T*^ is a contribution of the intra-subject variance propagated into the second level over the generalized inverse of the first level design matrix *X*^(1)−^. Therefore the denominator in Eq. (2) contains two contributions:

(4a) $$\sqrt{c^{T}\mathrm{var}\left\{\beta^{\left(1\right)}\right\}c}=\sqrt{c^{T}C^{\left(2\right)}c+c^{T}X^{\left(1\right)-}C^{\left(1\right)}X^{\left(1\right)-T}c}=\sqrt{\Sigma^{2}+\sigma^{2}}\text{,}$$

(4b) $$\mathrm{where}\;\Sigma^{2}=c^{T}C^{\left(2\right)}c\;,\;\mathrm{and}\;\sigma^{2}=c^{T}X^{\left(1\right)-}C^{\left(1\right)}X^{\left(1\right)-T}c\text{.}$$

In the following we refer to the contribution ∑^2^ as inter-subject variability and to the contribution *σ*^2^ as intra-subject variability. In order to estimate intra-subject variance *σ*^2^ additional fixed effects group level analyses for each sequence were performed. The maps of the intra-subject variance *σ*^2^ were calculated using Eq. 4b and estimate for *C*^(1)^ based on the maximal likelihood estimation of residual variance maps (ResML) resulting from the fixed effects group level analysis. The var{*β*^1^} variance maps were estimated based on the residual variance maps resulting from the second level random effects group analysis for each contrast. The maps of inter-subject variance ∑^2^ were then estimated using Eq. 3 by taking the voxel-wise difference between *cT* var{*β*^(1)^}*c* and *σ*^2^.

#### Regions of interest

Two region of interest (ROI) analyses were performed. The first ROI analysis was used to provide a descriptive comparison of the sensitivity of four sequences in multiple brain regions in fixed and random effect analyses. The second ROI analysis made statistical inferences about the relative sensitivity of the four sequences under investigation.

Several ROIs across the entire brain were defined in MNI standard space. Since the tasks included visual input, motor responses with the right hand, face processing and decision-making, the following five brain regions were expected to be activated in the contrast ‘tasks vs. rest’: bilateral primary visual cortex (V1), bilateral lateral geniculate nucleus (LGN), and left primary hand motor cortex (M1). In addition deactivation in the medial prefrontal cortex (MPFC) was expected, since MPFC is part of the default-mode network that is generally more active in resting conditions. One substantial difference between the EL and RBL tasks is the visual processing of faces and facial expressions in the EL task. Therefore, the following nine parts of the face-processing network were expected to be more strongly involved in the EL than in the RBL task and therefore activated in the contrast ‘EL vs. RBL’: fusiform face area (FFA), extrastriatal face area (EFA), amygdala, and orbitofrontal cortex (OFC) (Fusar-Poli et al., 2009). The fusiform place area (FPA) was hypothesized to be more strongly activated by the RBL task.

Three different strategies for ROI design were used to create nine ROIs in total. The first strategy defined the ROI a priori based on anatomical atlases. It was used to define ROIs in V1, M1 and OFC. The V1 ROI was extracted from the SPM anatomical toolbox (Eickhoff et al., 2005) using an anatomical ROI including Brodmann Area 17 (Amunts et al., 2000). The M1 and the OFC ROIs were defined as spherical ROIs with a diameter of 10 mm centered at the MNI coordinates (40 − 20 62) for the left M1 and (3 51 − 14) for the OFC.

The second strategy for ROI design was based on an intersection of activation clusters extracted from thresholded random effects functional activation maps obtained with the four sequences. A voxels was only included in the ROI if all four sequences detected an activation in it. This second strategy was applied to define ROIs in the bilateral LGN, bilateral FFA, bilateral FPA and bilateral EFA. Contrast ‘task vs. rest’ was used for LGN definition. Contrast ‘EL vs. RBL’ was used for FFA, FPA and EFA definition. A threshold at the uncorrected voxel-wise significance level of t = 5.5 was used, since it provided well-separated clusters for all four sequences.

The third strategy was used to obtain the ROI in the amygdala. The bilateral amygdalae were defined as an intersection of anatomical masks extracted from the SPM anatomical toolbox using the Amygdala label (Amunts et al., 2005) and four clusters obtained from the thresholded random effects activation maps provided by four sequences in the contrast ‘EL vs. RBL’. The threshold level of t = 5.5 was used.

For the descriptive ROI analysis the mean ROI values of group t-values obtained in fixed and random effects analyses, contrast values, inter-subject variability ∑^2^ and intra-subject variability *σ*^2^, were extracted from the corresponding maps by averaging over all voxels in ROI. These measures were obtained for the four EPI sequences and both contrasts.

In order to quantify the impact of intra-subject variance on the second level analysis, we also calculated the ratio of intra-subject to total second level variance $r=\frac{\sigma^{2}}{\Sigma^{2}+\sigma^{2}}$ for each voxel and each sequence. The mean ratio r averaged over all ROI voxels ${\langle r\rangle }_{ROI}={\langle \frac{\sigma^{2}}{\Sigma^{2}+\sigma^{2}}\rangle }_{ROI}$ were extracted for two contrasts for each sequence.

The second ROI analysis performed a formal statistical comparison of the sensitivity of the four sequences in random effect analysis. For this statistical ROI analysis the mean ROI contrast values *c^T^β*^(1)^ for each subject, two contrasts and four EPI sequences were extracted from the contrast maps obtained in fixed effects single subject analyses. For this purpose data without spatial smoothing were used. A one sample t-test, performed on mean ROI values over subjects, provided ROI t-values for two functional contrasts. In order to compare the sensitivity of the four sequences, we tested the null hypothesis assuming no difference in the expected t-values provided by the different sequences. Bayesian probability of this hypothesis was estimated by integration of a bimodal shifted t-distribution as it is described in the SI. Comparisons with p < 0.05, uncorrected for multiple comparisons, were regarded as significant.

## Results

### Group level analysis

**Fig. 2:**
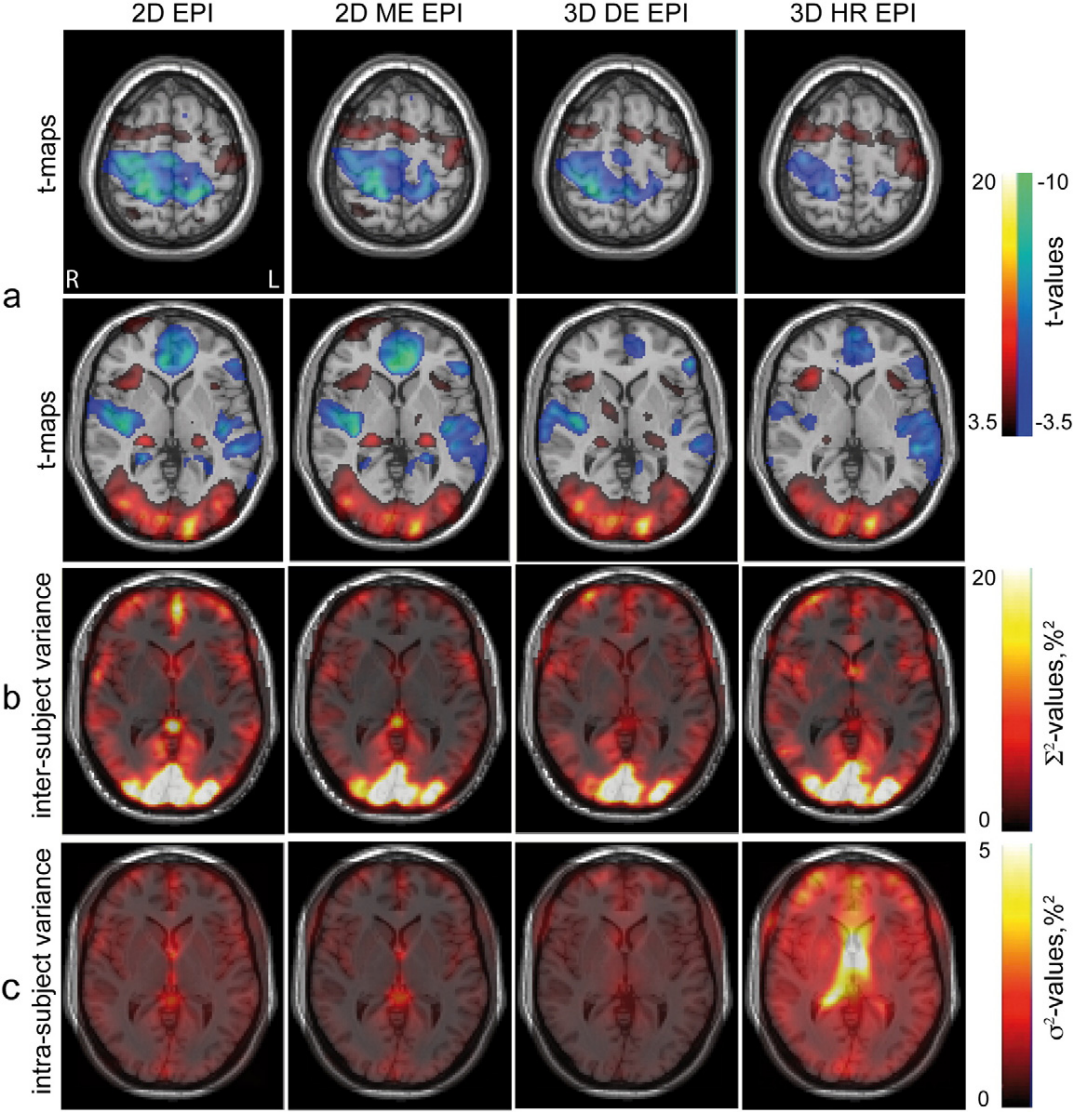
Cerebral activation group analyses for contrast ‘tasks vs. rest’ obtained with the four EPI sequences (columns 1 to 4). (a) Random effects activation t-maps overlaid on a single-subject *T*_1_-weighted image with the voxelwise significance threshold level p < 0.001 uncorrected for multiple comparisons. (b) Inter-subject and (c) intra-subject variance maps overlaid on a single-subject *T*_1_-weighted image.

Fig. 2 shows group level analysis results for the contrast ‘tasks vs. rest’. The upper row (Fig. 2a) presents activation t-maps resulting from a random effects analysis obtained for the four sequences overlaid on a single subject anatomical image. From Fig. 2a it becomes apparent that all four sequences provided similar activation maps in the random effects analysis for contrast ‘tasks vs. rest’. All sequences robustly detected activation in the primary visual, left primary motor, left somatosensory areas, and right insula, and deactivation in the MPFC. These main activation clusters for the contrast ‘tasks vs. rest’ are summarized in Table 2. In the visual cortex the 3D DE EPI sequence provided the highest peak t-values, in the motor cortex the 2D ME EPI. Deactivation in the MPFC was most reliably detected by 2D ME EPI. Activation in the bilateral LGN was detected by all sequences, but did not survive cluster level FWE-correction for 3D HR EPI. 2D EPI, 3D DE EPI and 3D HR EPI yielded significant activation in the superior temporal gyrus (STG). Activation in the left insula was significant for all sequences except 2D EPI. All clusters of significant activation are summarized in Table S1 of the supplementary information (SI).

**Fig. 3:**
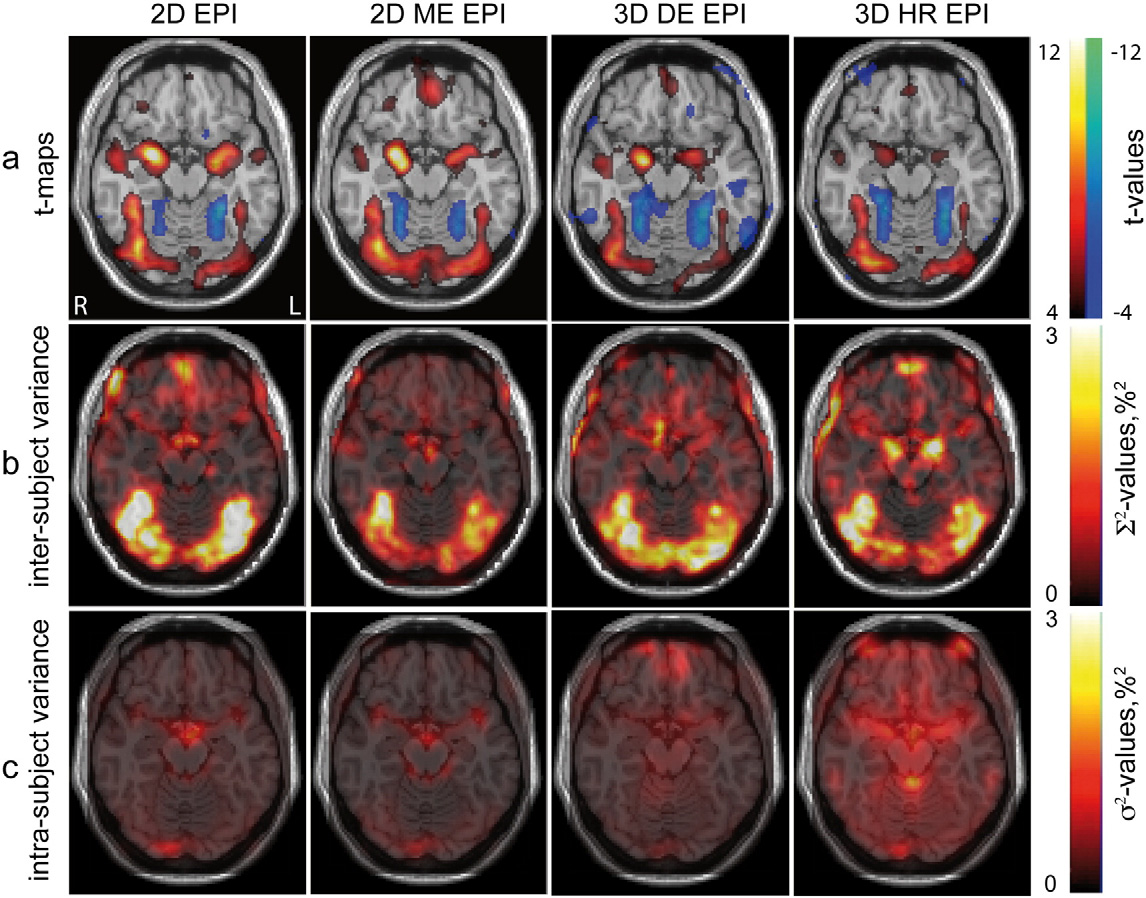
Cerebral activation group analyses for contrast ‘EL vs. RBL’ obtained with the four EPI sequences (columns 1 to 4). (a) Random effects activation t-maps overlaid on a single subject *T*_1_-weighted image with the threshold level p < 0.001 uncorrected for multiple comparisons. (b) Inter-subject and (c) intra-subject variance maps overlaid on a single subject *T*_1_-weighted image.

Fig. 3 shows group level analysis for the contrast ‘EL vs. RBL’. One can see in Fig. 3a that all sequences provided similar results in random effects analysis for this contrast. All sequences showed activations in the bilateral FFA, bilateral amygdala, bilateral EFA, and right medial frontal gyrus (MFG), as well as deactivation in the bilateral FPA and extrastriatal place area. These main activation clusters provided by each sequence are summarized in Table 3. 2D ME EPI and 3D DE EPI detected significant activation in the OFC. 2D ME EPI, showing the highest t-values and largest activation cluster most robustly detected activation in the OFC. An activation cluster in the left medial frontal gyrus (MFG) was detected by 2D EPI and 3D DE EPI. An activation cluster in the precuneus was detected by all sequences except 3D HR EPI. Standard 2D EPI yielded the highest t-values in the amygdala. All clusters of significant activations are summarized in Table S2 of the SI.

The maps of intra-subject variance *σ*^2^ for all four sequences and two contrasts are presented in Figs. 2c and 3c. The level of the intra-subject noise and its distribution across the brain were different for all four sequences. 2D EPI and 2D ME EPI showed similar spatial noise distributions but the noise amplitude found for 2D EPI was generally higher. For both 2D sequences higher noise amplitudes were observed in gray matter, around the circle of Willis, in the occipital and sagittal sinuses and close to the brain steam.

The 3D sequences showed higher intra-subject variance in comparison to the 2D sequences. The noise was more homogenously distributed over the gray and white matter. For 3D DE EPI higher noise levels were observed in the center of the brain. 3D HR EPI demonstrated the highest intra-subject noise, with higher levels in the vicinity of ventricles.

Inter-subject variance ∑^2^ for the contrasts ‘tasks vs. rest’ and ‘EL vs. RBL’ is shown in Figs. 2b and 3b, respectively. In contrast to intra-subject variability, which differs across sequences, inter-subject variability maps for both contrasts were very similar for the four sequences. Inter-subject variance showed strong differences across the brain with gray matter showing higher variance values. For the contrast ‘tasks vs. rest’ the highest variance was observed in the primary visual cortex (see Fig. 2b) and in the OFC. For contrast ‘EL vs. RBL’ the highest variance was observed in the fusiform gyrus. Both single echo sequences, 2D EPI and 3D HR EPI, showed higher amplitudes of the inter-subject variance in the OFC as compared to their multi-echo counterparts, 2D ME EPI and 3D DE EPI (see Fig. 3b). 3D HR EPI showed higher inter-subject variance in the center of the brain around the amygdala.

The final smoothness of the data as described by full-width at half maximum (FWHM) estimated for four sequences in fixed (random) effect analysis was estimated by SPM as: 9.4 mm (12.6 mm) for 2D EPI, 8.6 mm (13.8 mm) for 2D ME EPI, 8.1 mm (13.5 mm) for 3D DE EPI, and 6.3 mm (11.4 mm) for 3D HR EPI. Here we report the geometric mean of (FWHM) estimated for the x, y and z directions.

### Region of interest analysis

**Fig. 4:**
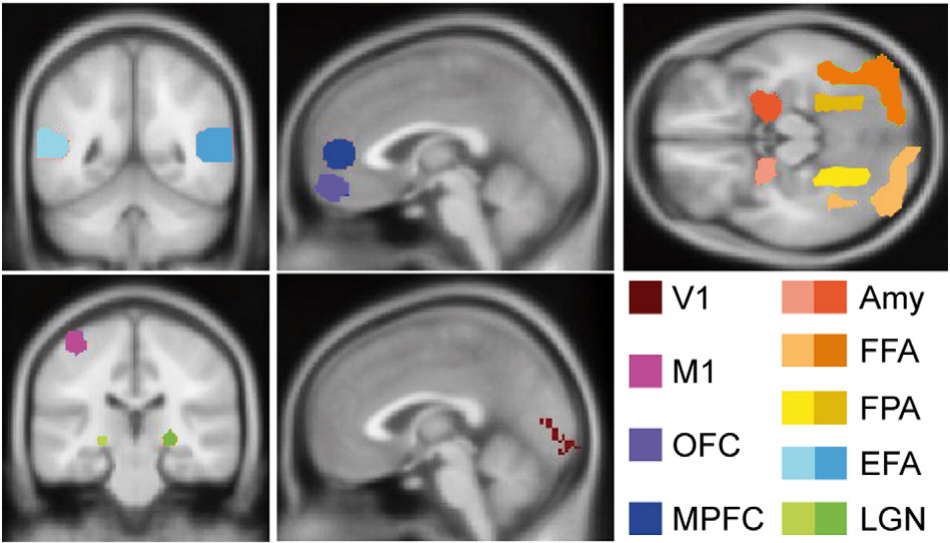
ROIs employed in the data analyses: Primary visual cortex (V1), primary left motor cortex (M1), orbitofrontal cortex (OFC), medial prefrontal cortex (MPFC), bilateral amygdala (Amy), bilateral fusiform face area (FFA), bilateral fusiform place area (FPA), bilateral extrastriatal face area (EFA) and bilateral lateral geniculate nucleus (LGN).

**Fig. 5:**
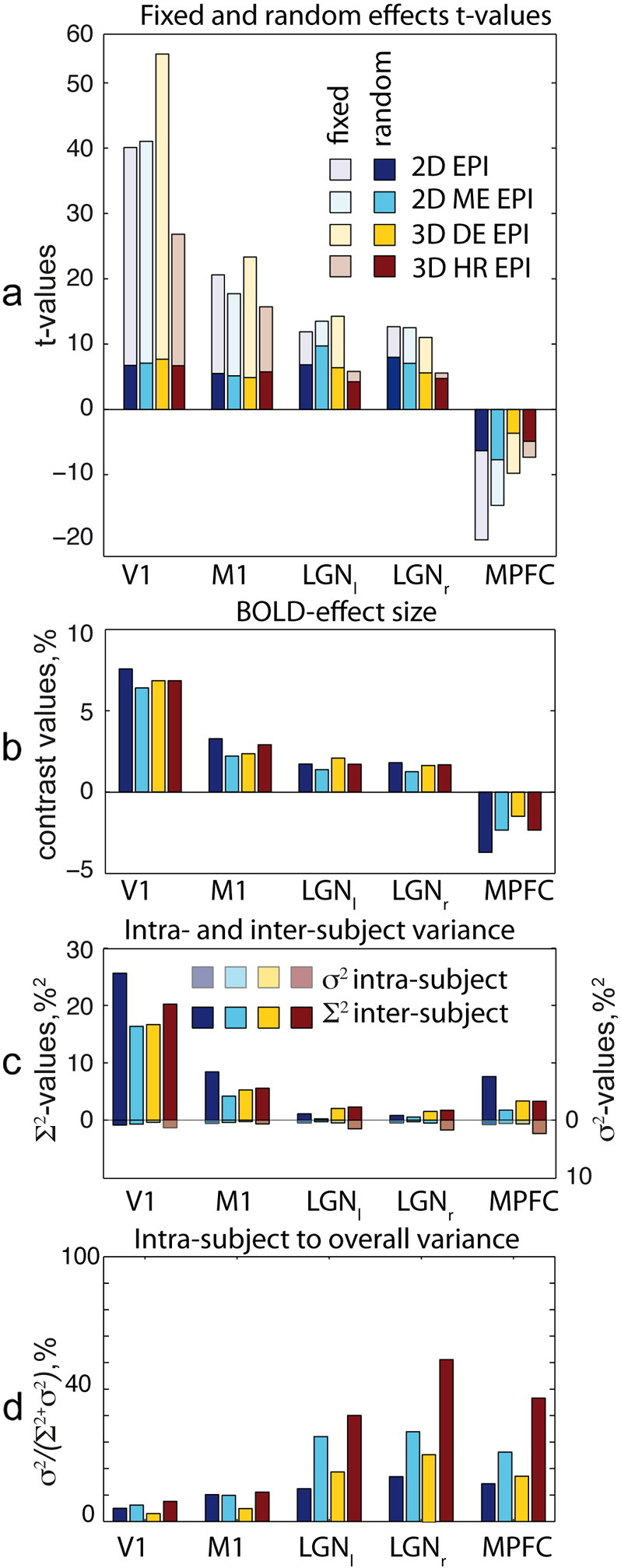
Cerebral activation ROI group analyses for contrast ‘tasks vs. rest’ (*c* = [1 1 1 1 1 1]) for the four EPI sequences and five ROIs. (a) t-Values resulting from fixed and random effects analyses. p-Values obtained for significant differences between two particular random effects t-values are given together with black bars spanning the respective t-values. (b) ROI-averaged contrast values, (c) inter-subject variance ∑^2^ and intra-subject variance *σ*^2^ averaged over ROIs. Please note the reversed axis direction for intra-subject variance *σ*^2^. The total length of the both colored bars combined corresponds to the sum of both variances and thus to the second level variance, (d) ROI means of the ratio between intra-subject to total random effects variance.

**Fig. 6:**
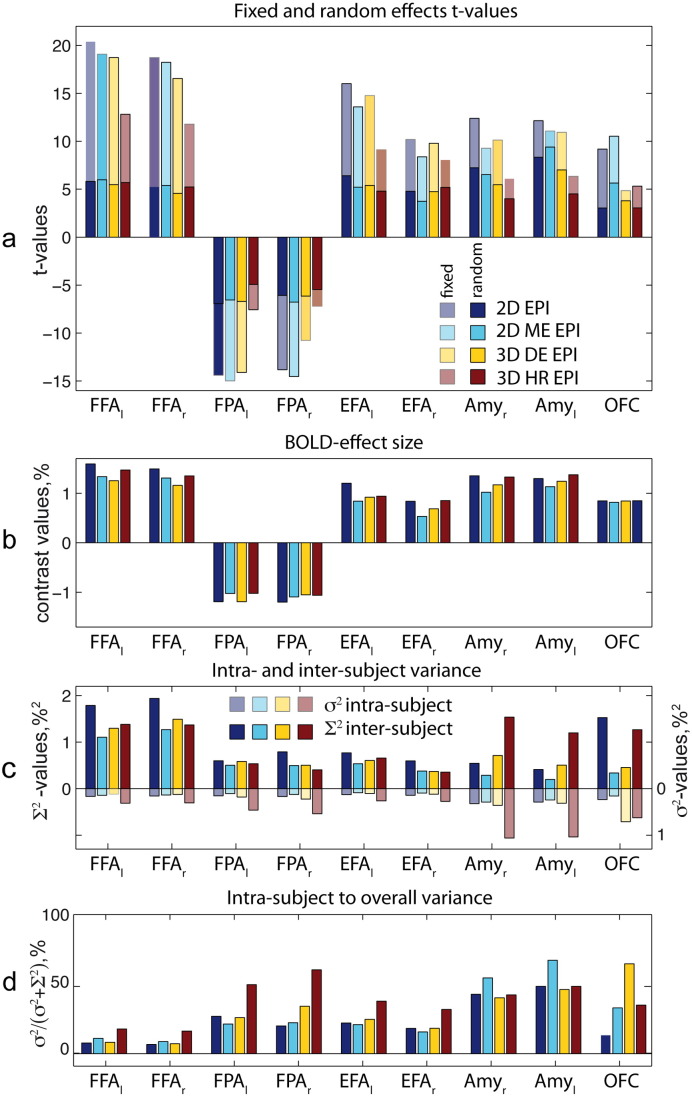
Cerebral activation ROI group analyses for contrast ‘EL vs. RBL’ (*c* = [1 1 1 − 1 − 1 − 1]) for the four EPI sequences and nine ROIs. (a) t-Values resulting from fixed and random effects analysis. p-Values obtained for significant differences between two particular random effects t-values are given together with black bars spanning the respective t-values. (b) ROI-averaged contrast values, (c) inter-subject variance ∑^2^ and intra-subject variance *σ*^2^ averaged over ROIs. Please note the reversed axis direction for intra-subject variance *σ*^2^. The total length of the both colored bars combined corresponds to the sum of both variances and thus to the second level variance, (d) ROI means of the ratio between intra-subject to total random effects variance.

The ROIs used in our analyses are presented in Fig. 4. The descriptive ROI analysis results for both contrasts are shown in Fig. 5, Fig. 6. Figs. 5a and 6a display mean ROI t-values for fixed and random effects analyses. Figs. 5b and 6b present contrast values averaged over ROI. Figs. 5c and 6c show inter- and intra-subject variances averaged over each ROI. Figs. 5d and 6d show the ratio of intra-subject to total variances averaged over each ROI. In the primary visual and motor areas for contrast ‘tasks vs. rest’ and in the FFA for the contrast ‘EL vs. RBL’ the inter-subject variance was much larger than the intra-subject variance. In these areas the intra-subject variance contributed less than 10% to the overall variance of the random effects analysis for all sequences except 3D HR EPI. As a result t-values originating from fixed effects analysis were much higher than those obtained from random effects analysis (see Figs. 5a and 6a).

A different situation was observed in the LGN and MPFC for the contrast ‘tasks vs. rest’ and in the amygdala and OFC for the contrast ‘EL vs. RBL’. In these areas intra-subject and inter-subject variances were comparable. The intra-subject contribution into the overall variance of random effects analysis is above 30% for these regions. As a result absolute fixed effects analysis t-values were higher, but not much different as compared to the random effect analysis results in all sequences.

In the statistical ROI analysis no significant difference in the random effects analysis ROI t-values was found between the four sequences in V1 and M1 areas for contrast ‘tasks vs. rest’. Also for contrast ‘EL vs. RBL’ no differences between sequence sensitivities in the FFA, EFA and left FPA were found. However, several following significant differences in sensitivity between the tested sequences were obtained. 3D HR EPI provided lower t-values as compared to the other sequences in several brain regions. 2D EPI provided significantly higher t-values than 3D HR EPI in the left FPA as well as in the right and left amygdala (p = 0.04, p = 0.028, p = 0.026 respectively). 2D ME EPI outperformed 3D HR EPI in the right amygdala and OFC (p = 0.003, and p = 0.03 respectively).

In addition we found that 2D ME EPI yielded better results than 2D EPI in the OFC with significantly higher t-values in this region (p = 0.022).

## Discussion

This study provides the first systematic comparison of functional sensitivities in fMRI population studies achieved with standard 2D EPI and advanced EPI sequences, including 2D ME EPI, 3D DE EPI and 3D HR EPI. Sensitivities at the group and single subject levels were assessed by fMRI during emotional and reward based learning, reliably activating a wide-spread network including the primary and secondary visual cortices, sensory–motor cortex, orbitofrontal cortex as well as subcortical areas.

For fixed effects analysis we found the following sensitivity differences for the tested EPI sequences to cerebral activation (see Figs. 5a and 6a). In the primary visual and primary motor areas 3D DE EPI clearly outperforms standard 2D EPI in the fixed effects analysis, providing higher t-values in these areas (see Fig. 5a). In deeper brain areas (e.g. FFA or amygdala), however, 3D DE EPI demonstrated comparable or slightly lower performance than standard 2D EPI (see Fig. 6a). 3D HR EPI yielded lower fixed effects t-values in comparison to the other sequences in all brain regions for both contrasts.

Sequence dependent differences in fixed effects t-values may be attributed to differences in the intra-subject variance which primarily originates from physiological and/or thermal noise (Triantafyllou et al., 2005). Multi-shot 3D sequences provide a higher signal to thermal noise ratio (Poser et al., 2010) but also accumulate physiological noise over the whole *T*_Rvol_ period — comparable with the periods of respiratory and cardiac pulsations.

Although a significant amount of noise can be removed by physiological noise correction, some of the remaining variance deteriorates the sensitivity achievable by the 3D sequences. Especially the 3D HR EPI with the slower *T*_Rvol_ of 2.5 s is affected by this effect. 3D EPI acquisitions were used for two specific applications – fast (*T*_Rvol_ = 1 s) and high resolution (2 mm) imaging – each involving a penalty in temporal SNR per volume and therefore in sensitivity to brain activation as compared to standard acquisitions. The fast 3D DE EPI implementation yielded fixed effect t-values similar to those of the slower acquisitions in cortical areas, where the SNR of modern receive coil arrays is largest. The penalty in sensitivity due to the high acceleration factor employed by both 3D sequences (AF = 2 × 3 and 2 × 2, respectively) was observed to be more prominent in deeper brain regions. This is consistent with the lower SNR and higher g-factor of receive coil arrays there. The use of 3D EPI acquisition schemes for high-resolution acquisitions allows for a reduction in voxel size at minimum cost in temporal SNR as compared to 2D EPI (Lutti et al., 2013, Poser et al., 2010). Recently reported improvements in temporal SNR (128% in visual cortex and 164% in LGN at 1.5 mm^3^ resolution) partly compensate the reduction in voxel size between the 3 mm and 2 mm resolution protocols used here (27 mm^3^ and 8 mm^3^ respectively). The lower t-values provided by the 3D HR EPI sequence in the fixed effects analysis are consistent with the reduction in voxel size, particularly in deep brain regions where the SNR provided by modern multi-channel coils is lowest.

Despite their different performances in fixed effects analyses, the random effects activation maps provided by the four tested sequences were strikingly similar (see Figs. 5a and 6a). Nearly in all brain regions comparable random effects analysis t-values were obtained. This can be attributed to the dominating contribution of inter-subject variability and highlights the possibility of increasing the acquisition speed or the image resolution at minimum cost in the second-level analyses. More generally speaking, our findings indicate that despite the performance gains obtainable with advanced EPI sequences in fixed effect analyses, standard EPI also provides similar t-values and therefore robust performance in random effects group level analyses. However, two exceptions from these general findings are identified.

First, we found lower sensitivity of 3D HR EPI in deep brain regions — LGN, FFA, FPA, amygdala and OFC. The principal SNR advantage of 3D acquisitions can only partly compensate for the loss in SNR due to the smaller voxel size. As a result lower statistical values were found in random-effect analyses for the high-resolution acquisitions, most likely due to dominating thermal noise components.

The second prominent exception is the superior performance of multi-echo sequences in orbitofrontal areas. This area is known to be affected by susceptibility-related drop-outs due to the vicinity of the air-filled frontal sinuses (Weiskopf et al., 2006, Weiskopf et al., 2007a, Deichmann et al., 2003). The short echo time images in the multi-echo acquisition and flexible adjustment of the multi-echo image combination allow for an effective recovery of signal in areas affected by susceptibility-related signal loss (Poser et al., 2006).

Outside the brain areas affected by susceptibility-related BOLD sensitivity loss, standard 2D EPI showed similar performance in most brain areas in random effects analysis as compared to the tested advanced EPI sequences. This finding is surprising in view of the superior performance of the advanced EPI sequences in the fixed effects analysis.

This observation may be explained by a comparison of intra-subject and inter-subject variance contributions to the random effects analysis. Comparing variance maps and ROI-variances in Fig. 2, Fig. 3, Fig. 5, Fig. 6, it becomes apparent that for all pulse sequences and all brain areas the inter-subject variance dominates the total variance in the random effects analysis. As a result random effects analysis t-values in these cases are much lower than the fixed effects analysis t-values. Thus, they are determined by properties of the subject population rather than by the sensitivity provided by a particular EPI sequence. For these brain regions further improvements of the acquisition methods do not necessarily translate into increased sensitivity in random effects analyses.

The only exception to the latter observations is random effects analysis results for 3D HR EPI and the amygdala and OFC, as explained above. In these cases the intra- and inter-subject variance contributions to the random effects analyses are comparable. Therefore, improvements in the single subject analysis are reflected in better performance on the random effects level.

The present comparative study is based on specific imaging parameters and a specific pre-processing pipeline for standard and advanced sequences. This particular choice of experimental parameters limits the validity of the general conclusions drawn here for some special cases. In fact, higher spatial resolution than the chosen 3 mm or smaller smoothing kernels would influence the thermal noise and thereby intra-subject variance contribution (Triantafyllou et al., 2006). Surface-based co-registration routines demonstrated reduced inter-subject variance (Tucholka et al., 2012) and could therefore provide improved group results.

Moreover using lower FA (Gonzalez-Castillo et al., 2011) or different repetition times would result in changes of the intra-subject noise and therefore different results. Thus, our results could not be generalized to the latter cases. However, our imaging parameters as well as the pre-processing pipeline are similar to the ones used in the majority of recent 3 T fMRI studies when focusing on group results, whole brain coverage and moderate resolution. Therefore, our results are relevant for a large proportion of state of the art fMRI applications.

One additional difference between the sequences when comparing group level analysis is difference in data smoothness.

For fixed effect analyses smoothness is dominated by an 8 mm smoothing kernel applied in the pre-processing step. Even in these cases, we found notable differences between the four sequences. Latter differences in data smoothness are not surprising taking into account the different acquisition schemes employed by the four sequences (2D vs. 3D and single echo vs. multiecho). The different schemes result in different physiological and thermal noise contributions (see Figs. 2c and 3c).

For random effect analyses FWHMs for all sequences were significantly larger than for fixed effect analyses. This indicates a strong impact of inter-subject anatomical differences on data smoothness (Mikl et al., 2008). Although theoretically, the four methods should be equally affected by inter-subject differences, there might be slightly different inter-subject contributions in practice. For example different contrasts in EPI images could result in different registration and normalization efficiencies for the four sequences (Gonzalez-Castillo et al., 2013). This would then lead to differences in the final data smoothness for the four sequences.

Different FWHMs for the four methods result in different correction factors applied for the correction of multiple comparisons. For example smaller FWHM for 3D HR EPI is reflected by an increased effective number of independent observables and therefore in stricter multiple comparison correction, if the correction is performed on the voxel-wise level. Likewise, the significance would be influenced by the data smoothness for corrections on the cluster level. The tested advanced fMRI sequences did not include recently proposed multi-band EPI sequences (Setsompop et al., 2012, Feinberg et al., 2010). These sequences allow for even higher temporal resolution, opening up the possibility for efficient filtering of physiological noise and significantly higher t-values in fixed effects analysis. However, increased temporal resolution and effectively reduced physiological noise in multi-band sequences are expected to mainly affect intra-subject variance. According to our results the dominating contribution in random effects group analyses is the inter-subject variance. Therefore, we expect multi-band sequences to perform comparably to standard EPI in random effects group analysis for whole brain coverage and the moderate spatial resolution used here.

Recently Gonzalez-Castillo et al. demonstrated that optimal fMRI sensitivity on the single subject level fixed effects fMRI analysis is not necessarily achieved by optimizing image SNR (Gonzalez-Castillo et al., 2011). The authors showed that for the most frequently used imaging parameters physiological noise dominates over thermal noise. Therefore, flip angles much smaller than the Ernst angle may be employed without a significant decrease in fMRI sensitivity. Our study goes one step further and demonstrates, that for the group level random effects group fMRI analysis intersubject variance dominates over physiological noise. As a result, improvements of the first level sensitivity do not necessarily translate into improved sensitivity on the second level.

The implications of the present study reach far beyond a mere performance test of four particular pulse sequences. Based on the obtained experimental results the following general guidelines for the fMRI sequence selection may be formulated. For random effects fMRI studies, where no a priori hypothesis about the area of interest is available or where multiple superficial and deep brain areas are targeted, standard EPI still provides a reasonable performance. For random effects fMRI in the OFC, ME EPI is the sequence of choice, due to its superior sensitivity in these areas. Furthermore, 3D DE EPI may provide advantages for primary sensory areas and when maximum sensitivity in the single subject activation maps is required. This might be the case when a functional localizer is included to obtain individual ROIs for each particular subject or in general real-time applications (Weiskopf et al., 2007b). In these cases the increased sensitivity in the fixed effects single subject level of 3D DE EPI is a clear advantage. 3D HR EPI should be chosen when optimum resolution in combination with whole brain analysis is required.

We identify the high level of inter-subject variability and its impact on the random effects analysis as a challenge for future fMRI method optimization. The sources of inter-subject variability are multifold. They include inter-subject differences in the underling cognitive processes, differences in cerebral anatomy and vascularization, scalp geometry, and physiological difference in the amplitude of the BOLD response. While some of these differences (e.g. variability underling psychological process) are intrinsic property of the studied population and are of particular interest for neuroscientist, all other sources of the inter-subject variance degrade fMRI sensitivity. Recently approaches were presented (Kalcher et al., 2013; Kazan et al., 2015) which allow for rescaling of single subject activation maps to account for inter-subject differences in BOLD response amplitudes. Our study indicates that these approaches have a high potential to further improve fMRI sensitivity in random effects group analysis.

## Conclusion

This study compared three advanced EPI sequences with conventional single shot 2D EPI, both in fixed effects as well as in random effects group analyses. In accordance with recent studies we confirmed that the tested advanced EPI sequences provide increased sensitivity at the single subject level. However, at the random effects group analysis level, when whole brain coverage and moderate resolution are used, the standard EPI sequence provided a sensitivity comparable to the advanced EPI sequences in most cases studied. We note activation studies of the orbitofrontal cortex affected by susceptibility artifacts as an important exception, since multi-echo EPI provides superior sensitivity as compared to their single echo counterparts. Their increased sensitivity in this region on the single subject level effectively translates into a higher sensitivity on the group level. This finding suggests that other methods reducing signal loss would also result in similar improvements (e.g., Weiskopf et al., 2007a, Weiskopf et al., 2006).

Our findings have implications that go beyond making the proper choice of a particular experimental parameter or sequence. We could show that optimization strategies for improved fMRI data acquisition need to focus more on reducing inter-subject variability. While improvements at the single subject level are achievable by optimized pulse sequences, there is a need for optimization strategies specifically targeting the sensitivity in second level group analysis. We anticipate that such optimizations may be best achieved by advanced data acquisition and post-processing methods.

## Figures and Tables

**Table 1:** Sequence parameters of the four EPI sequences used in this study.

Sequence	*T*_R_ [ms]/*T*_Rvol_ [ms]	*T*_E_ [ms]	FA	AF	Matrix/resolution	PF	BW [Hz/Px]
2D EPI	2000	30	70°	2 (A–P)	In plane 64 × 64 37 slices (20% gap) 3 × 3 × 3 mm^3^	No	2232
2D ME EPI	2500	7.4, 17.2, 27, 37, 47	70°	3 (A–P)	In plane 64 × 64 37 slices (20% gap) 3 × 3 × 3 mm^3^	6/8	2520
3D DE EPI	50/1000	15.9, 34.4	15°	2 × 3 (A–P) × (L–R)	Matrix 64 × 64 × 60 3 × 3 × 3 mm^3^	No	2367
3D HR EPI	70/2520	33	20°	2 × 2 (A–P) × (I–S)	Matrix 128 × 64 × 128 2 × 2 × 2 mm^3^	No	1408

2D EPI — 2D echo planar imaging; ME EPI — multi-echo EPI; DE EPI — dual echo EPI; 3D HR — 3D high resolution EPI; FA — flip angle; AF — acceleration factor; PF — partial Fourier factor; BW — bandwidth.

**Table 2:** Results of the random effects group analysis for contrast ‘tasks vs. rest’ obtained with the four EPI pulse sequences. All reported activation clusters were significant at an uncorrected voxelwise significance level of p < .001 and a cluster whole brain FWE-corrected level of p < 0.05 (for one or more sequences).

Brain region	2D EPI			2D ME EPI			3D DE EPI			3D HR EPI		
*N*_vox_	*T*_max_	*p*_FWE_	*N*_vox_	*T*_max_	*p*_FWE_	*N*_vox_	*T*_max_	*p*_FWE_	*N*_vox_	*T*_max_	*p*_FWE_
V_1_	7465	20.4	0.000	8008	16.5	0.000	8343	21.8	0.000	6152	19.14	0.000
Left M_1_ & left S_1_ Right insula	4015	8.3	0.000	4322	11.24	0.000	3183	10.11	0.000	2837	9.7	0.000
Left LGN	89	9.5	0.013	83	12.45	0.006	466^a^	7.38	0.000^a^	12	4.84	0.882
Right LGN	75	10.7	0.028	104	9.78	0.013	7.14	25	6.54	0.396

a Large clusters containing several structures were detected.

**Table 3:** Results of the random effects analyses for contrast ‘EL vs. RBL’ for the four pulse sequences. All reported activation clusters were bigger than 10 voxels. They were significant at an uncorrected voxelwise significance level of p < .001 and a cluster whole brain FWE-corrected level of p < 0.05. *N*_vox_ is a number of voxels in the cluster, *T*_max_ is a local maximum t-value, and *p*_FWE_ is a p-value after family wise correction on a cluster level.

Brain region	2D EPI			2D ME EPI			3D DE EPI			3D HR EPI		
*N*_vox_	*T*_max_	*p*_FWE_	*N*_vox_	*T*_max_	*p*_FWE_	*N*_vox_	*T*_max_	*p*_FWE_	*N*_vox_	*T*_max_	*p*_FWE_
Left amygdala	6548^a^	9.5^a^	0.000^a^	295	8.3	0.002	4256^c^	7.2^c^	0.000^c^	97	5.2	0.728
Right amygdala	6548^a^	15.3^a^	0.000^a^	882	14.2	0.000	4256^c^	11.8^c^	0.000^c^	100	6.4	0.089
Left FFA	6548^a^	8.0^a^	0.000^a^	4199^b^	8.2^b^	0.000^b^	4256^c^	6.9^c^	0.000^c^	881	7.5	0.007
Right FFA	6548^a^	10.2^a^	0.000^a^	4199^b^	11.2^b^	0.000^b^	4256^c^	10.7^c^	0.000^c^	1807^d^	10.3^d^	0.000^d^
Left EFA	344	5.8	0.283	65	4.3	0.969	4256^c^	6.1^c^	0.000^c^	213	6.1	0.000
Right EFA	6548^a^	12.2^a^	0.000^a^	4199^b^	8.6^b^	0.00^b^	4256^c^	8.8^c^	0.000^c^	1807^d^	10.26^d^	0.000^d^
OFC	46	4.5	0.977	644	7.6	0.007	94	5.7	0.283	28	4.9	0.865

^a,b,c,d^ Large clusters containing several structures were detected. The same cluster is indicated by the same letter.
