# Supplementary material for: The quest for the best: The impact of different EPI sequences on the sensitivity of random effect fMRI group analyses
Source: Neuroimage. 2016 Feb 1;126:49–59. doi: 10.1016/j.neuroimage.2015.10.071 (PMC4739510; doi:10.1016/j.neuroimage.2015.10.071)

# Supplementary Information

#
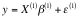
,


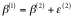
,

Where
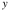
 is a time trace of voxel intensity measured with EPI sequence after de-meaning and de-trending,
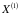
 is a design matrix of the first level fixed effects analysis containing effects of interest.
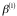
 and
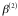
 are the first and second level effect size vectors.
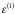
 is a first level error with intra-subject covariance
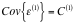
 and
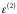
 is a second level error with inter-subject covariance
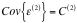
. Using the Eq.1 and 2 the second level mixed effects are obtained in terms of first level estimators using the generalized inverse of the first level design matrix
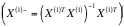
:


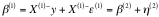
, where
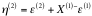


The covariance of the second level estimators is then given by
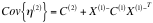
 and is a sum of the inter-subject covariance
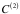
, and intra-subject covariance contribution projected through the inverse of the first level design matrix to the second level. Then for a particular contrast
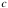
 of interest the t-value is provided by
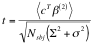
, where
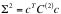
 is inter-subject variance contribution and
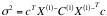
 is a contribution of the intra-subject variance into the second level.

Inference in fMRI studies is typically based on the Student t-test of the null-hypothesis that a certain brain region or certain voxel is not involved in the task processing, i.e., that the BOLD response does not differ from zero. In the fixed and mixed effects analysis the dimensionless t-value corresponds to the ratio of the amplitude of the BOLD response (or effect size) and its intra- and inter-subject variance (see Eq.2). Due to the different pulse sequence components and timing of the signal the four tested EPI sequences both effect size and variance are systematically different. For example, the multi-echo sequences (2D ME EPI and 3D DE EPI) combine images corresponding to different T_E_. Since the BOLD signal amplitude is proportional to T_E_ the effect size detected by these two sequences systematically differs from that obtained with the single echo sequences 2D EPI and 3D HR EPI. On the other hand the impact of physiological processes such as cardiac and respiratory cycles on the fMRI signal is also *T*_E_ dependent (Kundu et al. 2012). Moreover the distribution of physiological noise across the brain is different. Therefore, the a reasonable measure of the sequence performance needs to include effect size and variance. The statistical t-values fulfill this requirement.

The t-values estimated from fMRI data analysis are themselves stochastic variables. Thus, their comparison requires a statistical approach. In our study the comparison of t-values derived from the mixed effects analyses is complicated by the fact that only a single t-value is available for each EPI sequence and voxel or ROI. In the following, we derive a method for comparing two t-values based on a Bayesian model of the experimentally obtained t-values.

If we assume *t*_1_ and *t*_2_ to be t-values, resulting from a given mixed fMRI group effects analysis obtained on the same subject population of *N* (in our case *N*=24) subjects, but in two separate sessions with different pulse sequences, EPI_1_ and EPI_2_, respectively. In this case *t*_1_ and *t*_2_ are independent stochastic variables. Each of them has a non-central Student t-distribution *f*(*N,T*_nc1_*,t*_1_) and *f*(*N, T*_nc2_*, t*_2_) with *N* degrees of freedom and the non-centrality parameters *T*_nc1_ and *T*_nc2_, respectively. The expected values of *t*_1_ and *t*_2_ are then given by:

E(*t*_1,2_) ≈1.031**T*_nc1,2_ Eq.S1

A sensitivity comparison of two pulse sequences is equivalent to a comparison of their expected t-values, or equivalently their non-centrality parameters. The non-centrality parameters *T*_nc1_ and *T*_nc2_ are unknown, but some experimental realization of *t*_1_ and *t*_2_ are obtained experimentally. In this case the Bayesian posterior probability distribution for *T*_nc1,2_ is given by:


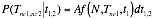
 Eq.S2

where A is normalization constant. Since *T*_nc1_ and *T*_nc2_ are independent, their joint probability density function is a product of the two probability density functions. Now, the Bayesian probability for the hypothesis that EPI_1_ is significantly more sensitive then EPI_2_ (or *T*_nc1_ > *T*_nc2_) is given by the experimentally obtained *t*_1_ and *t*_2_ values, which are achievable by 2D integration over *T*_nc1_ and *T*_nc2_, according to:


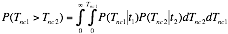
Eq.S3

The resulting probability function is shown in Fig S1.

**Table S1.** Mixed effects group analyses for contrast task vs. rest for the four pulse sequences. All reported activation clusters were bigger than 10 voxel and were found to be significant at an uncorrected voxelwise significance level of *p* < .001 and a cluster whole brain FWE-corrected level of *p* < 0.05.

| Brain region | 2D EPI | | | 2D ME EPI | | | 3D DE EPI | | | 3D HR EPI | | |
| --- | --- | --- | --- | --- | --- | --- | --- | --- | --- | --- | --- | --- |
|  | *N*_vox_ | *T*_max_ | *p*_FWE_ | *N*_vox_ | *T*_max_ | *p*_FWE_ | *N*_vox_ | *T*_max_ | *p*_FWE_ | *N*_vox_ | *T*_max_ | *p*_FWE_ |
| V_1_ | 7465 | 20.35 | 0.000 | 8008 | 16.5 | 0.000 | 8343 | 21.8 | 0.000 | 6152 | 19.14 | 0.000 |
| Left M_1_&Left S_1_&Medial Frontal Gyrus&RightMiddle Gyrus &Right Insula | 4015 | 8.29 | 0.000 | 4322 | 11.24 | 0.000 | 3183 | 10.11 | 0.000 | 2837 | 9.7 | 0.000 |
| Left LGN | 89 | 9.53 | 0.013 | 83 | 12.45 | 0.006 | 466 | 7.38 | 0.000 | - | - | - |
| Right LGN | 75 | 10.69 | 0.028 | 104 | 9.78 | 0.013 |  |  |  | - | - | - |
| STG | 110 | 9.03 | 0.004 |  |  |  | 94 | 6.26 | 0.023 | 964 | 6.45 | 0.019 |
| BA6 Frontal Lobe  (-51 2 34) | 72 | 5.72 | 0.033 |  |  |  |  |  |  |  |  |  |
| BA6 Frontal Lobe  (-15 68 -17) |  |  |  | 176 | 4.97 | 0.001 |  |  |  |  |  |  |
| left DLPFC |  |  |  | 75 | 4.01 | 0.049 | - | - | - | 111 | 6.23 | 0.001 |
| right DLPFC |  |  |  |  |  |  |  |  |  | 337 | 5.9 | 0.000 |
| left Insula |  |  |  | 88 | 6.51 | 0.026 | 78 | 6.26 | 0.047 | 85 | 6.75 | 0.005 |
| Globus Pallidus |  |  |  |  |  |  | 96 | 5.72 | 0.021 |  |  |  |
| SFG, BA 11 |  |  |  |  |  |  | 94 | 5.11 | 0.023 |  |  |  |

Table S2. Mixed effects analyses for contrast ‘EL vs. RBL’ for the four pulse sequences. All reported activation clusters were bigger than 10 voxel and were significant at an uncorrected voxelwise significance level of *p* < .001 and a cluster whole brain FWE-corrected level of *p* < 0.05.

| Brain region | 2D EPI | | | 2D ME EPI | | | 3D DE EPI | | | 3D HR EPI | | |
| --- | --- | --- | --- | --- | --- | --- | --- | --- | --- | --- | --- | --- |
|  | *N*_vox_ | *T*_max_ | *p*_FWE_ | *N*_vox_ | *T*_max_ | *p*_FWE_ | *N*_vox_ | *T*_max_ | *p*_FWE_ | *N*_vox_ | *T*_max_ | *p*_FWE_ |
| left amygdala | 6548* | 9.5 | 0.000 | 295 | 8.3 | 0.000 | 4256* | 7.2 | 0.000* | 97 | 5.2 | 0.001 |
| right amygdala |  | 15.3 |  | 882 | 14.2 | 0.000 |  | 11.8 |  | 100 | 6.4 | 0.001 |
| left FFA |  |  |  |  | 8.2 |  |  | 10.7 |  | 881 | 7.5 | 0.000 |
| right FFA |  | 10.2 |  |  | 11.2 |  |  | 6.9 |  | 1807* | 10.3 | 0.000 |
| left EFA | 344 | 5.8 | 0.000 |  | 4.3 |  | 171 | 6.1 | 0.000 | 213 | 6.1 | 0.000 |
| right EFA | * | 12.2 | * | - | 6.7 | - | 4256* | 8.8 | 0.000* | 1807* | 9 | 0.000 |
| OFC | - | 4.5 | - | 644 | 7.6 | 0.000 | 94 | 5.7 | 0.015 | - | 4.9 | - |
| right MFG | * | * | * | 463 | 6.8 | 0.000 | 566 | 8.39 | 0.000 | 400 | 6.9 | 0.000 |
| left MFG | 64 | 4.18 | 0.033 | - | - | - | 195 | 6.44 | 0.000 | - | - | - |
| Precuneus | 375 | 5.9 | 0.000 | 114 | 5.1 | 0.000 | 79 | 4.6 | 0.031 | - | - | - |
| left FPA | 504 | -10.7 | 0.000 | 443 | -10 | 0.000 | 437 | -11.5 | 0.000 | 187 | -7.4 | 0.000 |
| right FPA | 324 | -10 | 0.000 | 430 | -10.4 | 0.000 | 261 | -11.1 | 0.000 | 164 | -10.0 | 0.000 |
| left EPA |  |  |  | 1219 | -9.5 | 0.000 | 398 | -10.7 | 0.000 | 184 | -7.8 | 0.000 |
| right EPA |  |  |  | 292 | -11.9 | 0.000 | 1158 | -9.1 | 0.000 | 194 | -8.9 | 0.000 |

**Figure S1.** Probability function for the sensitivity comparison of the two hypothetical EPI sequences, EPI1 and EPI2, based on the measured values *t*_1_ and t_2_, for a mixed effects analysis with 24 subjects.

Bayesian probability of the expected t-values E[*t*_1_]> E[*t*_2_] is given in colour code as a function of the experimentally obtained *t*_1_ and *t*_2_ values. Upper left and lower right areas correspond to values where the sensitivities of the two sequences significantly differ from each other the significance level of p<0.05.

# Figure S1

#
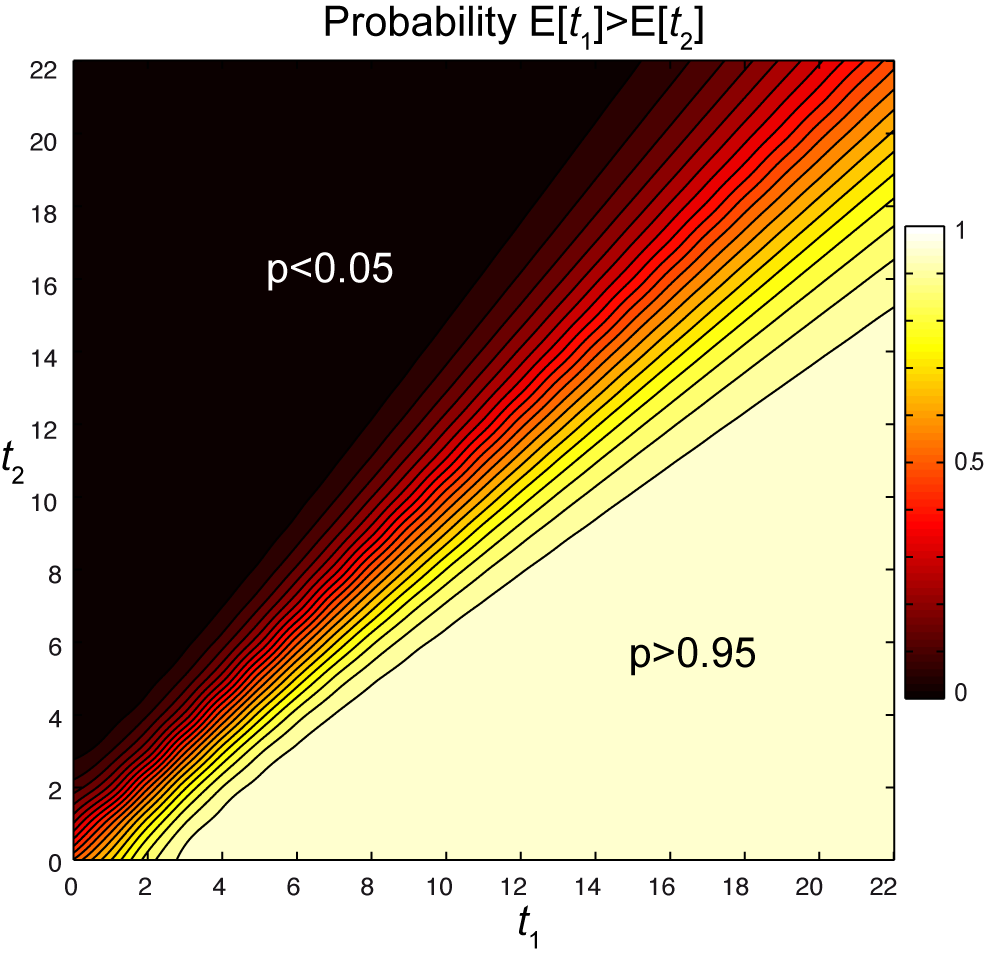

Supplement: Supplementary file 1 — Supplementary materials. [file mmc1.docx]
